# Exploring the spiritual needs of patients with advanced cancer in China: a qualitative study

**DOI:** 10.1038/s41598-024-54362-9

**Published:** 2024-02-18

**Authors:** Qinqin Cheng, Yongyi Chen, Yinglong Duan, Jianfei Xie, Qinghui Zhang, Hongling Zheng

**Affiliations:** 1 Nursing Department, Hunan Cancer Hospital/The Affiliated Cancer Hospital of Xiangya School of Medicine, Central South University, Changsha, China; 2 Nursing Department, The Third Xiangya Hospital, Central South University, Changsha, Hunan China; 3 Nursing Department, Sichuan Clinical Research Center for Cancer, Sichuan Cancer Hospital & Institute, Sichuan Cancer Center, Affiliated Cancer Hospital of University of Electronic Science and Technology of China, Chengdu, China

**Keywords:** Cancer, Health care

## Abstract

This qualitative study aimed to gain a deep understanding of the spiritual needs of patients with advanced cancer. A qualitative study using semi-structured interviews was conducted. The interviews were audio-recorded, transcribed verbatim, and subjected to thematic analysis. Two researchers coded the interviews independently in NVivo 12 plus and developed major themes and subthemes by inductive and constant comparison. This study was conducted in the inpatient ward of a tertiary cancer hospital in Hunan Province, Chinese Mainland. Eligible participants with advanced cancer were recruited using the purposive sampling method. The sample size was determined by data saturation. All interviews were conducted face-to-face individually from May 2021 to July 2021. A total of 13 patients with advanced cancer patients were interviewed. Six themes were identified, namely being treated as normal and independent individuals, receiving and giving love, seeking inner peace, connecting with spiritual sources, finding meaning and purpose, and preparing for death. Different categories of spiritual needs of patients with advanced cancer were identified in this study. Healthcare professionals need to develop interventions that aim to meet patients’ spiritual needs.

## Introduction

Spiritual care is beneficial for patients’ physical function and emotional, social, and spiritual well-being^1,2^, and it is a fundamental element of holistic nursing^3^. Cancer is a serious disease that is largely refractory and has a long course or treatment cycle. Throughout the cancer trajectory, patients with advanced cancer have experienced a variety of physical and psychological symptoms^4^. Patients’ spirituality is also seriously affected during the disease course. Spirituality is defined as a dynamic and fundamental component of humanity through which people search for ultimate meaning, purpose, and transcendence, as well as experience a relationship with the self, family, others, community, society, nature, and the meaningful or sacred^5^. Patients with advanced cancer have been shown to have lower levels of spiritual health than those with other diseases^6^. These patients often seek spirituality during the difficult course of their disease^7^, and almost every patient with cancer has at least one spiritual need^8^. Research has also reported that patients with cancer have a moderate need for spiritual care^9^. The prerequisite for providing high-quality spiritual care for patients is a deep understanding of their spiritual needs.

To date, there is no consensus on the definition of ‘spiritual need’. Mesquita et al.^10^ defined it as ‘the demand to connect with others, God, religion, well-being, and communication, as well as the desire to be regarded like a normal person’. Murray et al.^11^ described ‘spiritual need’ as humans’ innate expectations for the search for meaning, purpose, and worth in their lives. To date, an increasing number of studies have investigated the spiritual needs of patients with advanced cancer. Some researchers have used quantitative methods to explore the prevalence of spiritual needs^7,12–14^, while others have used qualitative interviews to gain deep insights into spiritual needs^15,16^. Different themes related to the spiritual needs of these patients have been extracted from various qualitative studies^15,16^.

Despite the considerable research on spiritual needs, few qualitative studies have focused on the spiritual needs of specific age groups^17^ or people with specific diseases^18^, and even fewer have focused on patients with advanced cancer. Moreover, most of the related studies have been conducted in Western countries. Therefore, little is known about the spiritual needs of patients with advanced cancer in the Chinese Mainland, where most people are irreligious^19^. As spiritual needs are influenced by one’s living environment, cultural background, and religious beliefs^20^, the spiritual needs of people in the Chinese Mainland may be different from those of people in other countries. Previously, we conducted a cross-sectional study to investigate the spiritual needs of patients with cancer using the Chinese version of the Spiritual Needs Scale^8^. However, this scale was originally developed in another society, which may have led us to overlook the needs specific to the Chinese Mainland population. Thus, the spiritual needs of Chinese patients with advanced cancer from their perspective warrant further exploration. The current qualitative study was conducted to fill this knowledge gap.

## Methods

This qualitative study used face-to-face, in-depth, semi-structured interviews to explore the spiritual needs of Chinese patients with advanced cancer.

### Participants and recruitment

This study was conducted at a tertiary cancer hospital in the Chinese Mainland from May 2021 to July 2021. The potential participants were patients who met the following inclusion criteria: (1) diagnosed with stage III or IV cancer; (2) aged 18 years or older; (3) physically able to participate; and (4) willing to provide informed consent to participate in this study. Those who had any mental illness and could not fluently communicate with the researcher were excluded. The participants were recruited using purposive sampling with maximum variation to ensure sample diversity in terms of age, sex, education level, marital status, and stage, diagnosis. The sample size was determined by data saturation when no new theme emerged from the interviews^21^. After interviewing 13 participants, considerable data were repeated, which meant that data saturation had been reached; thus, data collection was halted.

### Data collection

A semi-structured interview guide was developed based on the relevant literature and discussions among the research team members^22^. Questions related to spiritual needs, such as important things, inner needs, hope, the meaning of life, concerns, and wishes, were included in the interview guide. Face-to-face interviews were conducted individually by an oncology nurse researcher experienced in conducting interviews. The oncology nurse first approached eligible patients with advanced cancer in the inpatient ward and invited them to participate in this study after being informed of the purpose of the study. After obtaining their written informed consent, the oncology nurse made an appointment with them for the interviews. All of the interviews were conducted in a quiet, separate room of the hospital. Only the participant and researcher were present in the room during the interviews so that the participants could express themselves openly. All of the interviews were audio-recorded after informing the participants and obtaining their approval. The researcher encouraged the participants to express their feelings, thoughts, and experiences in the interviews without fear of interruption or judgment and took field notes to record the nonverbal cues, if any, during each interview. Each interview lasted 20–60 min.

### Data analysis

The interviewer and one researcher listened to the audio recordings repeatedly and transcribed them into text within 24 h of the interview to ensure transcript accuracy. One researcher, who was not the interviewer, conducted a preliminary analysis of the interview transcripts to determine when to stop the interviews based on data saturation. Data were analysed using thematic analysis, which involved several stages to systematically organise, reduce, refine, and ultimately analyse the data^23^. The interview transcript analysis was performed by two researchers independently using NVivo12 Plus software, which facilitates the management and analysis of qualitative data. Transcripts were analysed line-by-line and coded based on the questions of interest. Preliminary coding involved reviewing the whole dataset, highlighting any segments that contributed to the research question, and assigning initial descriptive labels to the emerging themes. After developing an initial coding scheme, researchers revised the coding scheme, recorded each transcript, and subsequently integrated them into a common coding scheme in joint data sessions^24^. Selective coding was applied to develop major themes and subthemes by using an inductive and constant comparison approach. Discrepancies between the two researchers were resolved through discussions with a third researcher until a consensus was reached. All research team members agreed with the results in a final discussion meeting^25^.

### Trustworthiness

The trustworthiness of this qualitative study was ensured by maintaining credibility, transferability, dependability, and conformability^26^. To ensure the credibility of this qualitative analysis, we adopted persistent observation, i.e., the researcher paying attention to the feelings or emotions of the interviewees^27^, to identify the most relevant information related to the participants’ experiences. We also routinely read and reread the data, analysed them, and revised the extracted concepts until the final abstracting provided an in-depth view of the participants’ experiences^28^. We attempted to describe the results thoroughly and clearly to enhance transferability^28^. To ensure dependability, we reported our process in detail so that other researchers could replicate the process if desired^29^. In addition, investigator triangulation was adopted by using two researchers to make coding, analysis, and interpretation decisions to develop more objective results and avoid misinterpretation or subjective imagination^30^.

### Statement

We confirm that all methods were performed in accordance with the relevant guidelines and regulations as the editor's office advised.

### Ethics declarations

This study was approved by Hunan Cancer Hospital (KYJJ-2020-141).

### Consent to participate/consent to publication

All participants consented to their anonymous data being published in peer-review journals during their informed consent.

## Results

A total of 13 patients with advanced cancer participated in this study. The median age was 50 years (range: 19–80 years). Table 1 shows the demographic characteristics of the participants. The interview data analysis yielded six themes about the spiritual needs of patients with advanced cancer: being treated as normal and independent individuals, receiving and giving love, seeking inner peace, connecting with spiritual sources, finding meaning and purpose, and preparing for death.

**Table 1:** Demographics of participants (*N*=13)

Variables	n	%
Age	Median: 50 (Range: 19–80)	
Sex		
Male	5	38.5
Female	8	61.5
Marital status		
Married	12	92.3
Unmarried	1	7.7
Education level		
High school or below	8	61.5
College or above	5	38.5
Type of cancer		
Cervical cancer	1	7.7
Sarcoma	2	15.4
Renal cancer	1	7.7
Rectal cancer	1	7.7
Malignant thymic carcinoma	1	7.7
Retroperitoneal sarcoma	1	7.7
Lung cancer	1	7.7
Liver cancer	1	7.7
Breast cancer	1	7.7
Colon cancer	1	7.7
Ovarian cancer	1	7.7
Gastric cancer	1	7.7
Cancer stage		
III	5	38.5
IV	8	61.5

### Theme 1: being treated as normal and independent individuals

#### Subtheme 1: maintaining normality

The participants expressed that due to their illness or their treatment, their physical function or self-image tended to change, which made them feel abnormal and caused psychological distress about not having a normal appearance. They stated that they tended to feel discomfort when other people did not treat them as normal people or showed them pity. They would like to be treated normally and act like normal people.

*After surgery, I lost one of my breasts. Normal women are beautiful (with two sides of the breast). However, I……(P9)*

*I am sick, and others are healthy, so I feel that I am not in the same world as them. (P5)*

*I do not want others to treat me as a patient; we used to play cards together. After I got sick, I felt that they were deliberately getting soft on me. I did not like this feeling. (P8)*

#### Subtheme 2: being independent

Due to the gradual decline in their physical function, patients with advanced cancer experience limitations in performing physical activities, including their daily self-care. However, they still wish to be able to perform their daily activities independently^31^. The participants stated that they tended to need their family members to take care of them with regard to daily activities, which elicited feelings of guilt for being a burden to their families. Thus, the patients wished to be independent so that they could take care of themselves and do not want to burden their family members. Accordingly, some of the participants expressed that they did not disclose their suffering to their family members so as to avoid burdening them.

*I hope that the function of my feet will return to normal so that I can walk on my own and live on my own. How I wish I could still drive to go on a sightseeing tour! No matter how hard it is! (P1)*

*I did not want to disturb my family when they were sleeping. It would be better if they got enough sleep when caring for me. They would feel uncomfortable if they did not sleep well and saw me in such pain. (P13)*

#### Subtheme 3: sustaining dignity

Some of the participants expressed experiencing both physical and psychological suffering during the course of the disease. Some even felt stigmatised by others who saw their suffering. Therefore, they stated that they preferred not to be visited by others in order to preserve their dignity.

*I do not want my friends to visit me. I know they are really worried about me, but I do not like their visits. (P8)*

*I do not want them to see my suffering; it is not good for them to see my suffering. I just want to retain a little dignity and do not want to be an object of pity to others. (P9)*

Some of the participants felt depressed due to the changes in their appearance caused by certain treatments. They expressed that they would like to maintain a good appearance in front of their loved ones.

*Sometimes, I saw my friends coming, and I did not want to see them because my appearance had changed significantly. (P11)*

### Theme 2: receiving and giving love

#### Subtheme 1: receiving support from loved ones

Some of the participants expressed that they had received tremendous support from their families, friends, or even acquaintances. They gained internal strength from such external support. Some stated that they needed care or companionship from their families.

*In my heart, what I need the most is care from my family. (P1)*

*Some time ago, I was not in a good mood; I know that was because I stayed for a long time in the hospital. So, I asked my son to come here, and I felt much better once I saw him. (P8)*

*I truly hope my mother can always accompany me. (P12)*

#### Subtheme 2: fulfilling family responsibilities

Due to their deteriorating physical condition, some of the participants, especially Chinese male participants, could not fulfil their original roles in their families. Men play an important role in traditional Chinese families and are known as the ‘pillars’ of the family, as they are responsible for physical work and providing for the family. Their declining physical condition weakened their family roles and made them feel useless. Some of the participants felt guilty for being unable to fulfil their family responsibilities, such as caring for the elderly or accompanying family members.

*My mother-in-law and father-in-law are in their 80s, and my mother has passed away. I wish I could leave the world after they all pass away so that I could fulfil my filial piety towards them. It would be regretful if I died before them. (P1)*

*Now I just want to relieve my suffering, see my granddaughter grow up a little bit, and accompany her longer. (P3)*

*I was in so much pain before I was hospitalised. I am a man and cannot do anything now. I truly wish I could help my family. (P7)*

#### Subtheme 3: helping others

In Chinese culture, there is a proverb, ‘Courtesy demands reciprocity’, which suggests that if someone helps you, you should try to help them back. One participant said that as she had received a lot of help and support from others, she would like to give back to others by helping them in any possible way. The participants expressed their willingness to continue to help others. In this way, they hoped to continue realising their value and extend love to others.

*I want to help others around me; then, I could feel like I am still useful to society. (P1)*

*I want to be nice to others before I die. I will try my best to do a good job and serve others. (P11)*

### Theme 3: seeking inner peace

#### Subtheme 1: sharing inner feelings

Although the participants did not want to increase their family’s burden, they stated that they would still like to seek support from others. Some of the participants expressed their need to share their inner feelings with their family members, especially when feeling discomfort.

*Actually, I do not want to wake them up. However, when I truly cannot stand it, I just want to share it with others. (P1)*

#### Subtheme 2: accepting disease

When they first learnt they had advanced cancer, most of the participants could not accept their diagnosis. Along their treatment journey, the participants gradually accepted their disease and usually attributed this ‘unfortunate’ event to fate. Some of the participants expressed that they had accepted their illness after going through a long phase of psychological reactions, while others still needed to persuade themselves to accept their disease.

*I thought it was just a little lump, but I did not know it was cancer. I had never thought about this. I cried for a few days! I cannot accept it. (P2)*

*Sometimes I kept thinking about why I got this disease. Why do I have such bad luck? (P10)*

#### Subtheme 3: sustaining positivity and hope

The participants expressed that they needed to sustain their hope and maintain positivity to support themselves. Since the participants had been diagnosed with life-threatening illnesses, they had realised that keeping healthy and living a happy life are the most important things. The participants stated that they had become aware that they did not have any other choice but to fight the illness to live longer.

*In my dreams, I always said to myself, ‘You must be tough; what else can you do if you are not tough?’ If I kept thinking of sad things, I would be unhappy. I could not eat well or sleep well. I am sick now. It is a fact that cannot be changed. Anyway, I need to keep an open mind and try my best not to overthink things. (P2)*

### Theme 4: connecting with spiritual sources

#### Subtheme 1: seeking folk beliefs

The participants expressed that they would like to turn to folk beliefs for spiritual support. The participants reported that they went to a temple to worship Buddha and practiced common religious activities such as burning incense, praying, or listening to Buddhist music. They thought that these activities could provide them with comfort and hope, especially during their difficult cancer journey.

*I worship Buddha…Worshipping Buddha has helped me a lot. I used to burn incense sticks, and this activity supported me for a few years. (P2)*

*I went to worship Buddha and prayed several times after I had the illness. (P10)*

*Sometimes, I would recite sutras and listen to Buddhist music. Doing this gave me a lot of strength. (P11)*

#### Subtheme 2: seeking inner faith

Some of the participants also described their need to seek inner faith despite their folk beliefs. Such inner faith can be derived from their inner will, a particular activity, or a special person. The participants expressed that they tried to find someone or something in which to have faith to support them and divert their attention from the disease. Such faith helped them gain the strength to survive the disease.

*My only motivation for survival is my daughter. She is the person who supports me to live longer. Umm…. I always woke up from my dreams at night, and a voice in my brain told me, ‘You have to fight the disease. There are so many things left to do.’ (P2)*

*I think my faith is practicing qigong; it supports me every day. I get up at approximately 5:00 am and practice qigong. (P10)*

### Theme 5: finding meaning and purpose

#### Subtheme 1: understanding the meaning of life

Before suffering from a life-threatening disease, few people think about death and the meaning of life. However, when their life is threatened by a disease, individuals may reflect on the meaning of life. In this study, some of the participants reported thinking about the meaning of life and finding an answer to live a meaningful life.

*Now I feel that I must keep a healthy body, only this; other things can make sense. Otherwise, nothing makes any sense. (P11)*

*Sometimes when I feel tired, I just ask myself, ‘What do you struggle for?’ (P9)*

#### Subtheme 2: realising wishes

The participants expressed that they still had unrealised wishes. They hoped to be able to realise these wishes even if they were in poor physical condition. Their wishes were mainly related to their families. If their wishes were fulfilled, they may pass away without regrets.

*I wish I could see my brother’s second child. If my brother has another child, she or he could be a substitute for me in my family. (P1)*

*I still have hope. I just want to see my son grow up and get married soon. (P8)*

### Theme 6: preparing for death

#### Subtheme 1: accepting death

The participants’ physical conditions sometimes deteriorated even though they received supportive care. As a result, they felt they had no other choice but to accept death. Some of the participants expressed that they needed to accept their imminent death, then they would have no regrets and die peacefully.

*I do not have any burdens. I would be delighted if I could die peacefully. (P5)*

*Unlike others, I am not afraid of death. I am not worried that I cannot get up anymore. I have no regrets. (P13)*

#### Subtheme 2: dealing with post-death issues

The death of an individual places a great psychological burden on their family. The family needs to arrange the affairs related to the funeral. In addition, other family issues may arise, such as inheritance and caring for children or the elderly. To not burden their families and avoid family conflicts, some of the participants expressed that they needed to arrange their personal affairs for after their death, such as financial issues, funerals, graves, and support for the elderly and children in the family.

*Now I have arranged all of the things in my home and for my funeral. I have already chosen my grave, spoken with my family members about everything, and made my wills. After doing these things, I feel relieved and can die peacefully. (P5)*

*If I die, my wife will have all my property, including my money, house, and car. I also told my brother to help take care of my family. I also told my wife that I would like to be cremated after I die. (P8)*

## Discussion

This study aimed to explore the spiritual needs of patients with advanced cancer from the first-person perspective. In this study, we have interviewed 13 patients. In general, based on a previous study^32^, about 12 informants were sufficient to generate rich data for addressing the question of interest in a qualitative study. Actually, after interviewing 13 participants, considerable data were repeated in the present study, which meant that data saturation had been reached. Therefore, comprehensive information regarding the spiritual needs of patients with advanced cancer had been obtained in this study. The findings provide deep insights into the spiritual needs of Chinese patients with advanced cancer, which may guide the development of culturally tailored interventions to provide high-quality spiritual care for these patients.

Our study found that patients with advanced cancer would like to be treated as normal and independent individuals. This finding is similar to that of a previous study^33^. It has been generally recognised that a majority of patients are eager to be involved in their own medical decisions as much as possible^34^. Being independent empowers them by giving a sense of control and self-esteem. In a previous study, one fourth of the participants reported that they needed to sustain their self-esteem (interpreted as ‘saving face’ in Chinese) by concealing their diseases or avoiding others worrying or gossiping about them^35^. In traditional Chinese culture, most people are eager to keep themselves in good shape. When they meet friends or visitors, they strive to make a good impression and ensure that their guests receive the best hospitality. Therefore, patients who suffer from serious illnesses do not want others to see them looking ill and are unwilling to be the object of excessive attention and kindness from others^36^. Accordingly, some of our participants stated that they were reluctant to be visited by friends and cause them concern. This finding suggests that healthcare providers should not show excessive compassion to patients with serious illness, but rather treat them as normal patients in the clinical setting.

Another need expressed by the participants was receiving and giving love. A previous study also found this need among Euro-American patients with cancer^37^. Family members and friends play an increasingly crucial role in meeting the spiritual needs of patients^18^. Patients would like to receive support, strength, and comfort from their family and friends, especially if they are socially isolated. Fulfilling their responsibilities and helping others may help patients feel that they are still useful and valuable^31^. Chinese people tend to have a strong sense of responsibility towards their families and believe the ancient Chinese proverb ‘courtesy demands reciprocity’, which means that if someone gives you love and support, you repay them by doing the same for them. Therefore, Chinese people consider receiving and giving love equally important.

The third theme identified in this study was seeking inner peace. For patients with advanced cancer, sharing their emotions contributes to having a good death^38^. Therefore, healthcare professionals could provide opportunities for these patients to share their feelings. When confronted with such a serious disease as cancer, patients tend to think about ‘why this bad thing happened to them’^39^. The disease, related treatment, and the imminence of death cause them distress^40^. Talking about death is considered taboo in Chinese culture because it is thought to bring misfortune. Therefore, when patients realise that their death is imminent, they believe that their only choice is to suppress their fear and distress. They are caught in an internal dichotomy of feeling both the fear of talking about death and an eagerness to share their suffering. Thus, accepting death is a challenging process for them^39^. Accordingly, our participants expressed their need to accept their disease. Although the disease journey was distressing, the participants expressed that it was important for them to sustain positivity and hope. Research has shown that keeping an open mind and happy thoughts and seeing others smile help patients to live in the moment and fight their illness^31^.

Connecting with spiritual sources was another important theme identified in this study. A previous study indicated that belief is an essential element for meeting spiritual needs in Chinese culture^41^. In this study, we found that patients with advanced cancer sought strength in two ways, namely through folk beliefs and inner faith. Religious faith is crucial for many of those who suffer from serious diseases^42^. However, most of the mainland Chinese population is irreligious, while some people seek folk beliefs for spiritual support. Meanwhile, some patients may find that certain people, things, or even inner thoughts serve as their inner faith and, consequently, a source of spiritual support. Both religious beliefs and inner faith tend to significantly impact patients and can help to guide their actions. Therefore, it is essential for healthcare providers to help patients with advanced cancer seek spiritual support from which they can gain strength to fight their illness.

The fifth theme was finding meaning and purpose. Maintaining a sense of life meaning and purpose is an essential aspect of spiritual care^43^. The present study found that the participants mainly wanted to understand the meaning of their own lives and realise their wishes. An individual’s sense of the meaning of life is influenced by a range of factors, including culture^44^. In Chinese culture, people prioritise their families and have a great sense of responsibility towards them. Healthcare providers may help patients think about the meaning of their life and their unfinished wishes through reviewing their lives, which may further help them realise their wishes.

Preparing for death was another major need mentioned by patients with advanced cancer, which was also observed in a previous study^37^. In this study, the participants cited preparing for post-death issues as an essential element of preparing for death. This finding is understandable as Chinese patients do not like to burden their families even in death. Hence, they may address such post-death issues ahead of time, such as making decisions about graves, funeral arrangements, and wills for property distribution. To address patients’ need to prepare for death, healthcare professionals and the patients’ families could talk about death-related issues with the patients. However, as death is a taboo topic in Chinese culture, talking about the topic is deemed ‘ominous’ and causes discomfort. Thus, healthcare professionals should explore appropriate ways to talk about death with their patients to help meet their needs related to preparing for death.

### Study limitations

This qualitative study provides an in-depth understanding of the spiritual needs of patients with advanced cancer in the Chinese context. However, the findings must be interpreted in light of some limitations. First, the spiritual needs of patients are influenced by their regional culture and environment; the participants involved in this study were all from one province. Therefore, the findings may not be generalisable to populations in other regions in China. Second, this study only interviewed inpatients; thus, it may not entirely reflect the spiritual needs of outpatients living at home.

## Conclusion

Different categories of spiritual needs of patients with advanced cancer were identified in this study, namely being treated as normal and independent individuals, receiving and giving love, seeking inner peace, connecting with spiritual sources, finding meaning and purpose, and preparing for death. As these needs are influenced by culture, healthcare professionals should develop culturally tailored interventions that aim to meet the spiritual needs of patients with advanced cancer.

### Supplementary Information

Supplementary Information.

## Data Availability

The datasets generated and analysed during the current study are not publicly available due to participants’ privacy but are available from the corresponding author on reasonable request.

## References

[1] Batstone E, Bailey C, Hallett N (2020). Spiritual care provision to end-of-life patients: A systematic literature review. J. Clin. Nurs..

[2] Cheng Q, Zhang Q, Liu X, Chen Y (2021). Initial exploration of training for palliative care specialist nurses in mainland China. Nurse Educ. Today.

[3] Selby D, Seccaraccia D, Huth J, Kurppa K, Fitch M (2017). Patient versus health care provider perspectives on spirituality and spiritual care: The potential to miss the moment. Ann. Palliative Med..

[4] He Y (2022). Symptom burden, psychological distress, and symptom management status in hospitalized patients with advanced cancer: A multicenter study in China. ESMO Open..

[5] Garcia ACM, Schneiders M, da Mota KS, da Conceição VM, Kissane DW (2023). Demoralization and spirituality in oncology: an integrative systematic review. Supportive Care Cancer Off. J. Multinatl. Assoc Supportive Care Cancer..

[6] Mohebbifar R, Pakpour AH, Nahvijou A, Sadeghi A (2015). Relationship between spiritual health and quality of life in patients with cancer. Asian Pac. J. Cancer Prev..

[7] Höcker A, Krüll A, Koch U, Mehnert A (2014). Exploring spiritual needs and their associated factors in an urban sample of early and advanced cancer patients. Eur. J. Cancer Care.

[8] Cheng Q, Xu X, Liu X, Mao T, Chen Y (2018). Spiritual needs and their associated factors among cancer patients in China: A cross-sectional study. Supportive Care Cancer.

[9] Yao, l. (2019). Meaningful Therapy Investigation and Intervention Study on Spiritual Care Needs of Advanced Cancer Patients.

[10] Mesquita AC, Chaves ÉCL, Barros GAM (2017). Spiritual needs of patients with cancer in palliative care: an integrative review. Curr. Opin. Supportive Palliat. Care.

[11] Murray SA, Kendall M, Boyd K, Worth A, Benton TF (2004). Exploring the spiritual needs of people dying of lung cancer or heart failure: A prospective qualitative interview study of patients and their carers. Palliat. Med..

[12] Astrow AB, Kwok G, Sharma RK, Fromer N, Sulmasy DP (2018). Spiritual needs and perception of quality of care and satisfaction with care in hematology/medical oncology patients: A multicultural assessment. J. Pain Symp. Manag..

[13] Gabriel I, Creedy D, Coyne E (2021). Quality of life and associated factors among adults living with cancer and their family caregivers. Nurs. Health Sci..

[14] Taylor EJ (2006). Prevalence and associated factors of spiritual needs among patients with cancer and family caregivers. Oncol. Nurs. Forum.

[15] Köktürk Dalcali B, Kaya H (2022). Spiritual care needs of patients in oncology units and nursing practices in turkey: A qualitative study. J. Relig. Health.

[16] Voetmann SS, Hvidt NC, Viftrup DT (2022). Verbalizing spiritual needs in palliative care: A qualitative interview study on verbal and non-verbal communication in two Danish hospices. BMC Palliat. Care.

[17] Cai S (2020). Spiritual needs and communicating about death in nonreligious theistic families in pediatric palliative care: A qualitative study. J. Palliat. Med..

[18] Chen HC (2019). The spiritual needs of community-dwelling older people living with early-stage dementia-a qualitative study. J. Nurs. Scholarsh..

[19] Chen Y, Zhao Y, Wang Z (2020). The effect of religious belief on Chinese elderly health. BMC Public Health.

[20] Memaryan N, Rassouli M, Mehrabi M (2016). Spirituality concept by health professionals in Iran: A qualitative study. Evid. Based Complement. Altern. Med..

[21] Guest G, Namey E, Chen M (2020). A simple method to assess and report thematic saturation in qualitative research. PLoS One.

[22] Kallio H, Pietilä AM, Johnson M, Kangasniemi M (2016). Systematic methodological review: Developing a framework for a qualitative semi-structured interview guide. J. Adv. Nurs..

[23] Braun V, Clarke V (2006). Using thematic analysis in psychology. Qual. Res. Psychol..

[24] Kiger ME, Varpio L (2020). Thematic analysis of qualitative data: AMEE Guide No. 131. Med. Teach..

[25] Gale NK, Heath G, Cameron E, Rashid S, Redwood S (2013). Using the framework method for the analysis of qualitative data in multi-disciplinary health research. BMC Med. Res. Methodol..

[26] Lincoln YS (1985). Naturalistic inquiry.

[27] Cope DG (2014). Methods and meanings: Credibility and trustworthiness of qualitative research. Oncol. Nurs. Forum..

[28] Korstjens I, Moser A (2018). Series: Practical guidance to qualitative research. Part 4: Trustworthiness and publishing. Eur. J. General Pract..

[29] Shenton AK (2004). Strategies for ensuring trustworthiness in qualitative research projects. Educ. Inform..

[30] Farmer T, Robinson K, Elliott SJ, Eyles J (2006). Developing and implementing a triangulation protocol for qualitative health research. Qual. Health Res..

[31] Edwards A, Pang N, Shiu V, Chan C (2010). The understanding of spirituality and the potential role of spiritual care in end-of-life and palliative care: A meta-study of qualitative research. J. Palliat. Med..

[32] Guest G, Bunce A, Johnson L (2006). How many interviews are enough?: An experiment with data saturation and variability. Field Methods.

[33] Vilalta A, Valls J, Porta J, Vinas J (2014). Evaluation of spiritual needs of patients with advanced cancer in a palliative care unit. J. Palliat. Med..

[34] Mesquita AC, Chaves ÉCL, Barros GAM (2017). Spiritual needs of patients with cancer in palliative care: An integrative review. Curr. Opin. Supportive Palliat Care.

[35] Hsiao SM, Gau ML, Ingleton C, Ryan T, Shih FJ (2011). An exploration of spiritual needs of Taiwanese patients with advanced cancer during the therapeutic processes. J. Clin. Nurs..

[36] Taleghani F, Yekta ZP, Nasrabadi AN (2006). Coping with breast cancer in newly diagnosed Iranian women. J. Adv. Nurs..

[37] Taylor EJ (2003). Spiritual needs of patients with cancer and family caregivers. Cancer Nurs..

[38] Bovero A (2020). Definition of a good death, attitudes toward death, and feelings of interconnectedness among people taking care of terminally ill patients with cancer: An exploratory study. Am. J. Hosp. Palliat. Care.

[39] Maiko S (2019). Spiritual experiences of adults with advanced cancer in outpatient clinical settings. J. Pain Sympt. Manag..

[40] Grant E (2004). Spiritual issues and needs: perspectives from patients with advanced cancer and nonmalignant disease. A qualitative study. Palliat. Supportive Care.

[41] Wu Q, Yin Y, Wang Q, Wang S, Jia X (2021). Body image and hopelessness among early-stage breast cancer survivors after surgery in China: A cross-sectional study. Nurs. Open.

[42] Stewart WC, Adams MP, Stewart JA, Nelson LA (2013). Review of clinical medicine and religious practice. J. Relig. Health.

[43] Elliott R, Wattis J, Chirema K, Brooks J (2020). Mental health nurses' understandings and experiences of providing care for the spiritual needs of service users: A qualitative study. J. Psychiatr. Mental Health Nurs..

[44] Karakas S, Okanli A (2014). The relationship between meaning of illness, anxiety, depression, and quality of life for cancer patients. Collegium Antropol..

