# Supplementary material for: Exploring the spiritual needs of patients with advanced cancer in China: a qualitative study
Source: Sci Rep. 2024 Feb 18;14:4009. doi: 10.1038/s41598-024-54362-9 (PMC10874944; doi:10.1038/s41598-024-54362-9)
Supplement: Supplementary file 1 — Supplementary Information. [file 41598_2024_54362_MOESM1_ESM.docx]

**Exploring the spiritual needs of patients with advanced cancer in China: A qualitative study**

Qinqin Cheng^1^; Yongyi Chen^1^; Yinglong Duan^2^; Jianfei Xie^2^; Qinghui Zhang^1^; Hongling Zheng^3^*

^1^ Hunan Cancer Hospital/The Affiliated Cancer Hospital of Xiangya School of Medicine, Central South University, Changsha, China

^2^ The Third Xiangya Hospital of Central South University, Changsha, Hunan, China

^3^ Sichuan Clinical Research Center for Cancer, Sichuan Cancer Hospital & Institute, Sichuan Cancer Center, Affiliated Cancer Hospital of University of Electronic Science and Technology of China, Chengdu, China

* Corresponding author: Hongling Zheng, Nurse Practitioner, MD, Nursing Management, Sichuan Clinical Research Center for Cancer, Sichuan Cancer Hospital & Institute, Sichuan Cancer Center, Affiliated Cancer Hospital of University of Electronic Science and Technology of China, Chengdu, China ([1375463713@qq.com,+8613696207051](mailto:1375463713@qq.com,+8613696207051))

Qinqin Cheng, Nurse Practitioner,PhD,[qin192323@outlook.com](mailto:qin192323@outlook.com)

Yongyi Chen, Professor of Nursing, PhD, 1722911690@qq.com

Yinglong Duan, Supervisor Nurse,MD,yinglongduan@outlook.com

Jianfei Xie, Associate Professor of Nursing,PhD,xiejianfei007@163.com

Qinghui Zhang, Nurse Practitioner, MSc, zhangqinghui@hnca.org.cn

Hongling Zheng, Nurse Practitioner,MD,1375463713@qq.com

**Appendix 1 Interview Guide**

1. What do you think is the most meaningful in your life after the illness?

2. What are your innermost needs?

3. What are the most important things for you at present?

4. What are your biggest concerns at present?

5. Do you have any wishes? If yes, would you like to share them?
